# Maintenance and assessment of cell viability in formulation of non‐sporulating bacterial inoculants

**DOI:** 10.1111/1751-7915.12880

**Published:** 2017-12-04

**Authors:** Teresa Berninger, Óscar González López, Ana Bejarano, Claudia Preininger, Angela Sessitsch

**Affiliations:** ^1^ AIT Austrian Institute of Technology GmbH Center for Health and Bioresources Bioresources Unit Konrad‐Lorenz‐Straße 24 3430 Tulln Austria

## Abstract

The application of beneficial, plant‐associated microorganisms is a sustainable approach to improving crop performance in agriculture. However, microbial inoculants are often susceptible to prolonged periods of storage and deleterious environmental factors, which negatively impact their viability and ultimately limit efficacy in the field. This particularly concerns non‐sporulating bacteria. To overcome this challenge, the availability of protective formulations is crucial. Numerous parameters influence the viability of microbial cells, with drying procedures generally being among the most critical ones. Thus, technological advances to attenuate the desiccation stress imposed on living cells are key to successful formulation development. In this review, we discuss the core aspects important to consider when aiming at high cell viability of non‐sporulating bacteria to be applied as microbial inoculants in agriculture. We elaborate the suitability of commonly applied drying methods (freeze‐drying, vacuum‐drying, spray‐drying, fluidized bed‐drying, air‐drying) and potential measures to prevent cell damage from desiccation (externally applied protectants, stress pre‐conditioning, triggering of exopolysaccharide secretion, ‘helper’ strains). Furthermore, we point out methods for assessing bacterial viability, such as colony counting, spectrophotometry, microcalorimetry, flow cytometry and viability qPCR. Choosing appropriate technologies for maintenance of cell viability and evaluation thereof will render formulation development more efficient. This in turn will aid in utilizing the vast potential of promising, plant beneficial bacteria as sustainable alternatives to standard agrochemicals.

## Introduction

Agricultural plant production is the basis for food, feed and fibre industry and thus plays a central role in supplying goods for our daily lives. However, meeting the demands of the growing global population proves challenging in face of climate change and the occurrence of crop pests and diseases, which frequently result in severe yield losses. Applying agrochemicals helps improving agricultural productivity, but often poses a risk to the health of farmworkers and consumers and has a negative impact on ecosystems (van der Werf, 1996; Damalas and Eleftherohorinos, 2011). The use of synthetic fertilizers – commonly referred to as NPK fertilizers – leads to the exploitation of limited phosphorus resources, nitrate pollution of groundwater and eutrophication of aquatic ecosystems (Conley *et al*., 2009). Moreover, its production requires a high energy input in form of fossil fuels (Kliopova *et al*., 2016).

In view of these drawbacks, sustainable approaches, such as the utilization of plant beneficial microorganisms, are becoming increasingly important.

### Beneficial mechanisms in plant–bacteria interactions

The most prominent example of a naturally occurring, beneficial interaction between plants and bacteria is the association of legumes with rhizobia. These bacteria reduce atmospheric nitrogen to ammonia and provide this essential nutrient to their host. Rhizobia on peat carriers have been commercially produced and knowingly delivered to the field to enhance soil fertility since the late 19th century (Brockwell and Bottomley, 1995). Since then, many other beneficial plant–bacteria interactions have been described (Compant *et al*., 2005; Glick, 2012; Ahemad and Kibret, 2014). These include the increase of nutrient availability by sequestering iron or other micronutrients, solubilizing phosphate from soil or fixing nitrogen. Furthermore, the tolerance to pests or pathogens is enhanced by inducing systemic resistance or controlling pathogens by out‐competition and antagonism. In addition, plant vigour may be enhanced by the production of phytohormones, modulation of phytohormone levels or detoxification of deleterious compounds. These effects may not only be conferred by rhizosphere‐dwelling bacteria, but also by those with an endophytic lifestyle (Rosenblueth and Martínez‐Romero, 2006; Hardoim *et al*., 2008, 2015; Ryan *et al*., 2008; Bhattacharyya and Jha, 2012). Considering the broad range of beneficial mechanisms, the modulation of the plant microbiota is a promising way to improving plant performance and ultimately agricultural production. This fact has spurred the exploration of microorganisms, mostly derived from rhizo‐ or endosphere, to be applied for the purpose of boosting crop production.

### The role of formulation in the utilization of plant‐associated bacteria

In many cases, results obtained under laboratory or glasshouse conditions are not easily transferred to the field, particularly when dealing with Gram‐negative, non‐sporulating bacteria. As they do not form spores, they are more susceptible to deleterious factors occurring during processing and field application (Potts, 1994; O'Callaghan, 2016). Therefore, they require suitable, protective formulations to enhance their efficiency at the target site and to facilitate the practical use by farmers. Several authors have provided comprehensive reviews on formulation development (Catroux *et al*., 2001; Malusá *et al*., 2012; Herrmann and Lesueur, 2013; Bashan *et al*., 2014; O'Callaghan, 2016). Nevertheless, a lack of adequate formulations and the concomitant low inoculant quality is still regarded as one of the major constraints to the successful, widespread use of microbial inoculants (Stephens and Rask, 2000). The delivery of a high number of viable cells to the plant is a prerequisite to reach a satisfactory colonization rate, which in turn enhances the desired effect in the field. The viability of inoculants may suffer at different stages before and during application. First, a product has to display a sufficiently long shelf life, which describes the stability throughout the production process, packaging, storage and transport conditions (Arora *et al*., 2011). During subsequent application on the field, the inoculant is confronted with additional factors that are detrimental to its viability. These include UV radiation (Zohar‐Perez *et al*., 2003), particularly when applied on above‐ground plant parts, fluctuating soil properties such as texture, temperature and pH (Arora *et al*., 2011) and repeated drying‐rewetting cycles depending on the frequency of precipitation. For inoculants applied directly to seeds, the inherent seed coat toxicity can be harmful (Deaker *et al*., 2012). Furthermore, biotic interactions with the native microflora and microfauna present a major challenge to any applied strains. Frequently, cell numbers of an introduced strain decline after application to non‐sterile soil as they are out‐competed by indigenous microbes or diminished by predators such as protozoa (Bashan, 1998; Arora *et al*., 2011). Obviously, pre‐application stress factors occurring during the production process are exacerbating this problem: the lower the number of viable cells delivered to the field, the less likely is a successful establishment at the target site. A mild formulation process is therefore of vital importance.

### Formulation technology: state of the art

**Figure 1:**
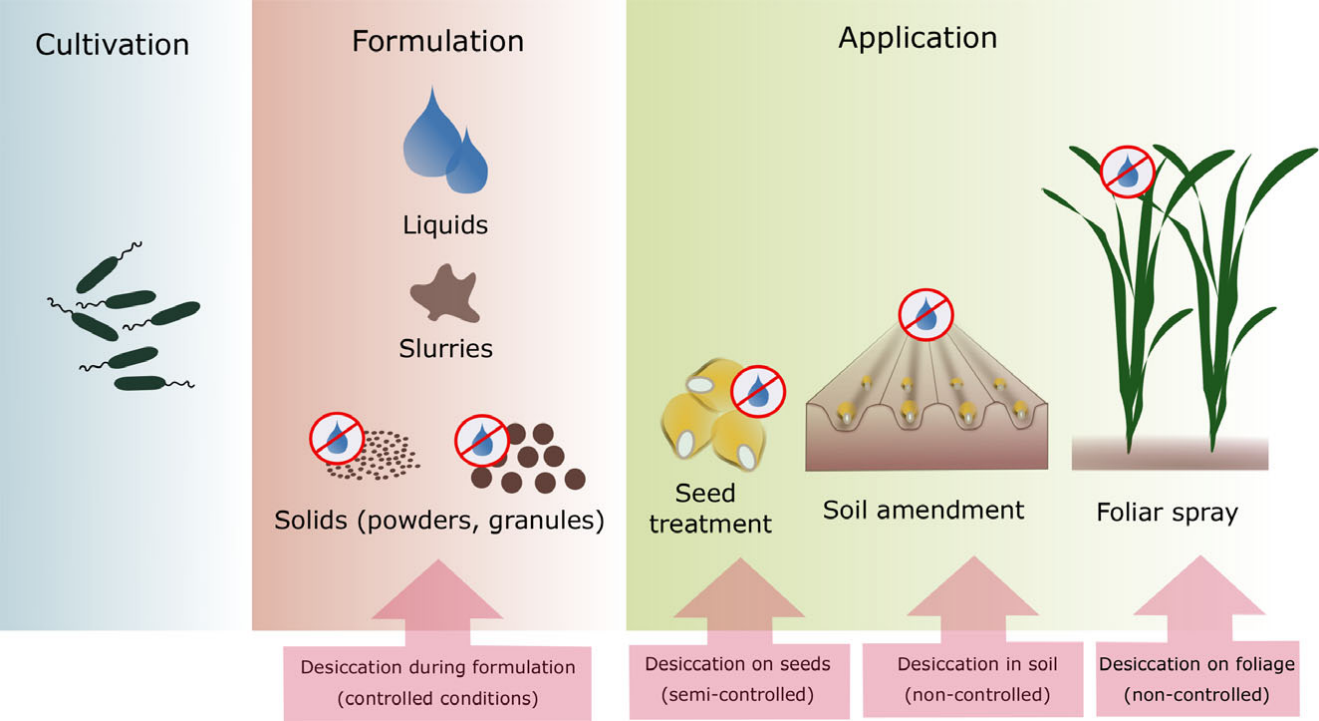
Formulation possibilities and inherent application techniques. Solids and slurries may be applied as seed treatment or soil amendment; liquids as seed treatments, soil amendment or foliar spray. The possibility of controlling the desiccation process depends thus on the form of the inoculant and application technique.

As in the case of standard agrochemicals, microbial products are formulated as solids, liquids or slurries (Fig. 1). Solid formulations may be subdivided into powders and granules depending on their particle sizes. In general, they are applied as seed coatings or soil amendments (Bashan *et al*., 2014). Apart from peat as a standard carrier material for dry formulations, several other options have been investigated (reviewed by Bashan *et al*., 2014; Malusá *et al*., 2012). These include soil‐derived carriers (e.g. charcoal, clays, turf), organic carriers (e.g. sawdust, wheat/soy/oat bran, grape bagasse, vermicompost, animal manure, sewage sludge, cork compost) and inert materials (e.g. perlite, vermiculite, bentonite, kaolin, silicates, talc, polymers). Regarding the latter, encapsulation of inoculant cells in polymers (e.g. alginate) has been proposed as a technique to ensure controlled release into soil (Dommergues *et al*., 1979; Bashan, 1986). In recent years, encapsulation technologies greatly advanced and have been employed to produce microbial inoculants varying in morphology and composition (reviewed by John *et al*., 2011; Schoebitz *et al*., 2013). Finally, pure lyophilized cultures, where desired in the presence of a lyoprotectant, may also be an option and can be used directly or in combination with a solid carrier (Malusá *et al*., 2012).

Alternatively, liquid formulations have been developed, comprising oil‐ or water‐based suspensions of cell concentrates, emulsions or slurries containing solid particles (Malusá *et al*., 2012). Additives such as protectants, nutritive substances, stabilizers or adhesives are often incorporated (Bashan *et al*., 2014). Liquids are suitable for a wide range of application technologies. Like solid formulations, they may be coated directly onto the seed (where applicable together with an adhesive) immediately prior to sowing (Bashan *et al*., 2014). Liquids may also be delivered to the soil in‐furrow during sowing or at a later stage via fertigation systems (Malusá *et al*., 2012). The latter technique is particularly relevant for the inoculation of perennial crops, where beneficial microorganisms need to be introduced into already established orchards or plantations (Malusá *et al*., 2012). Furthermore, liquids permit the treatment of above‐ground plant parts, for example in form of a foliar spray (Jambhulkar *et al*., 2016). This may be desirable if the active agent targets aerial plant parts (e.g. stomata, flowers) as entry ports for colonization, as has been observed for endophytic bacteria such as *Paraburkholderia phytofirmans* (Mitter *et al*., 2017). Similarly, the colonization strategy of plant pathogens and thus the site of action of a biocontrol strain may require its above‐ground application, as is the case, for example when controlling fire blight in the family of *Rosaceae* plants (Nuclo *et al*., 1998; Pusey, 2002).

Thus, the choice of the formulation technology depends on the application technique, available equipment, farmer's convenience, presence or absence of additional treatments, plant inherent characteristics (seed size, perennial/annual crop production, seed coating sensitivity), plant developmental stage, cost, site of action and colonization pathway of the inoculant (Deaker *et al*., 2004; Malusá *et al*., 2012; Bashan *et al*., 2014). To give some concrete examples of formulation approaches, Table 1 provides a non‐exhaustive list of commercially available biostimulants and biopesticides based on non‐sporulating bacteria along with their respective product characteristics.

**Table 1:** Examples of commercially available biostimulant and biopesticide products based on non‐sporulating bacteria

Product/manufacturer	Strain/viable cell count as specified by manufacturer	Distribution form and carrier specifications	Application techniques	Storage temperature and shelf life
Biostimulants				
BIODOZ^®^ Soja/Novozymes, Denmark	*Bradyrhizobium japonicum* /10^9^ per g	Wettable solids (peat based)	Slurry in water: seed coating	0 to 25°C, best before date (BBD) on package
BIODOZ^®^ Soja M /Novozymes, Denmark	*Bradyrhizobium japonicum* /10^9^ per g	Wettable solids (peat based) plus microgranules	Slurry in water and coating of microgranules: in‐furrow use	0 to 25°C, BBD on package
Cell‐Tech^®^ Liquid Soya bean/Monsanto BioAg™, Belgium	*Bradyrhizobium japonicum*/2 × 10^9^ per g	Liquid (aqueous carrier not specified)	Direct use: seed coating; diluted in water: in‐furrow use	Cool, 2 years
HISTICK^®^ Soy/BASF SE, Germany	*Bradyrhizobium japonicum*/2 × 10^9^ per g	Wettable solids (peat based), available with extra sticking agent NPPL^®^ Force 48 (polymer‐based)	Dry or slurry in water: seed coating	5°C, BBD on package
K Sol B^®^/Agri Life, India	*Frateuria aurantia* /5 × 10^7^ per g	Wettable powder (talc, dextrose, lignite)	Slurry with sugar and water: seed coating; slurry with manure and water: seedling root dip; mix with compost: soil amendment; dispersion in water: soil amendment via drip stream	No storage specifications, 1 year
Nitragin^®^ Gold Alfalfa and Sweetclover/Monsanto BioAg™, Belgium	*Sinorhizobium meliloti*/3 × 10^8^ per g	Micron‐sized particles (kaolin, bentonite, quartz)	Direct use: seed dusting	4 to 13°C, 2 years
NITROFIX™ – AC /Agri Life, India	*Azotobacter chroococcum*/5 × 10^7^ per g	Wettable powder (kaolin, dextrose, lignite)	As for K Sol B^®^	No storage specifications, 1 year
P Sol B^®^ PS/Agri Life, India	*Pseudomonas striata*/5 × 10^7^ per g	Wettable powder (talc, dextrose, lignite)	As for K Sol B^®^	No storage specifications, 1 year
Rizoliq^®^ Top S plus Premax^®^/Rizobacter, Argentina	*Bradyrhizobium japonicum*/2 × 10^9^ per ml	Liquid concentrate + separate liquid protectant (cellulose, disaccharides, nutrients)	Mix inoculant and protectant prior to use: seed coating	< 20°C, 2 years
Salavida/Sourcon Padena, Germany	*Pseudomonas trivialis*/amount not specified	Wettable powder (90% skimmed milk powder)	Dispersion in water: soil amendment, seedling root dip	−18 to 4°C, 8 months
Biopesticides				
Cedomon^®^/Lantmännen BioAgri AB, Sweden	*Pseudomonas chlororaphis*/10^9^ to 10^10^ per ml	Emulsion (rape seed oil)	Direct use: seed coating	4 to 8°C: 8 weeks; room temperature: 3 weeks
Cerall^®^ (Lantmänne BioAgri AB, Sweden)	*Pseudomonas chlororaphis*/10^9^ to 10^10^ per ml	Flowable suspension (water‐based)	Direct use: seed coating	4 to 8°C, 8 weeks
Galltrol^®^‐A (AgBioChem, USA)	*Agrobacterium radiobacter*, 1.2 × 10^11^ per plate	Bacterial culture on agar plates	Cell suspension in water: spray, root dip, root drench	2 to 4°C, 120 days
Sheathguard™ (Agri Life, India)	*Pseudomonas fluorescens*, 10^8^ per g	Wettable powder (carboxymethylcellulose, talc, cells)	Slurry with sugar and water: seed coating; Mix with manure or compost: nursery bed treatment, soil application	No storage specifications, 1 year

Regardless of the application form, desiccation represents a major bottleneck in formulation development. In solid formulations, drying occurs under controlled conditions during the technological processing, whereas liquid formulations face desiccation under semi‐controlled conditions (e.g. warehouse) when applied to the seed, or under non‐controlled environmental conditions after field application (Fig. 1). Furthermore, repeated drying‐rewetting cycles depending on precipitation and/or irrigation concern both solid and liquid inoculants after field application. Drying is often critical for sensitive organisms such as non‐sporulating bacteria and is regarded as one of the main reasons for their loss of viability both prior and after field application. Finding ways of alleviating the negative effects of desiccation is thus an essential step in enhancing the efficiency of bacterial products in the field.

Our review will therefore focus on the mitigation of drying stress in formulation development of non‐sporulating, agriculturally relevant bacteria. The key aspects we consider are the choice of the drying method, external addition of protectants, pre‐drying stress adaptation, application of bacterial exopolysaccharides and formulation with ‘helper’ strains. Moreover, we discuss methods for the assessment of cell viability and storage stability, including standard colony counting, spectrophotometry, real‐time quantitative polymerase chain reaction with propidium monoazide treatment (PMA‐qPCR), microcalorimetry as well as accelerated shelf life testing. Including these instruments in formulation development will speed up the process, provide high‐quality inoculants and ultimately contribute to the successful implementation of sensitive, but very promising plant beneficial bacteria.

## The drying process

Drying of microorganisms has been recognized as an efficient way of long‐term preserving. During desiccation of an organism – a state also known as anhydrobiosis – its vital functions come to a complete or partial halt and a state of dormancy is acquired. Upon rehydration, the organism is resuscitated and resumes its vital functions (García, 2011). In addition to favouring a high shelf life, dry products also reduce the costs associated with storage and distribution under refrigeration and are less prone to contamination (Meng *et al*., 2008). In establishing efficient and cheap drying protocols, the method itself, set‐up and matrix have to be considered (Prakash *et al*., 2013). This is an issue, for example in food industry when stabilizing probiotic bacteria for dietary intake and in pharmacy or research when conserving reference strains. Similarly, it is relevant for biocontrol agents (Morgan *et al*., 2006; García, 2011). However, despite the prospect of long‐term preservation of bacterial cells, the drying process itself often leads to a pronounced initial drop in the bacterial viability. Therefore, the bacterial survival rate is one of the main quality parameters to consider when screening for adequate drying protocols.

A range of drying methods has been explored in food industry when formulating probiotic bacteria, and the insights may well serve as references for agriculturally relevant bacteria. The most commonly used methods are freeze‐drying, vacuum‐drying, spray‐drying, fluidized bed‐drying and air‐drying, all of which differ in their mode of action and consequently the product characteristics they result in (García, 2011).

### Freeze‐drying

Freeze‐drying (lyophilization) essentially consists of two processing steps: pre‐freezing and sublimation of water by exposing the sample to high vacuum conditions. Sublimation describes the phase transition of the sample from solid to vaporous state and is dependent on its temperature and the surrounding vacuum. Below a certain value, which depends on the sample composition, a phase transition occurs immediately from solid (ice) to vaporous state without passing a liquid phase. The omission of melting renders the process a rather mild one and helps maintaining product characteristics. The final outcome of the sublimation process is influenced by the pre‐freezing temperature, pressure in the drying chamber, input temperature, amount of sample, end‐point of drying and instrument properties. This implies that there are countless combinations of process parameters to be evaluated when optimizing a freeze‐drying protocol for a given sample (Morgan *et al*., 2006). Drawbacks of lyophilization are the high costs (Santivarangkna *et al*., 2007) and the limited volume of this batch‐type operation (Morgan *et al*., 2006). Nevertheless, freeze‐drying is one of the most frequently applied methods in the formulation development of bacteria on laboratory scale. It has been evaluated for conservation of different strains of *Pseudomonas fluorescens* (Jean‐Noël *et al*., 2012; Cabrefiga *et al*., 2014; Bisutti *et al*., 2015), *Pseudomonas* spp. (Stephan *et al*., 2016) and strains of *Pantoea agglomerans* (Costa *et al*., 2000, 2002b; Soto‐Muñoz *et al*., 2015). Typically, the viability, e.g., of *Pseudomonas* spp. subjected to freeze‐drying was reduced by one to two orders of magnitude when no protective measures were undertaken (Stephan *et al*., 2016).

### Vacuum‐drying

Similarly to freeze‐drying, vacuum‐drying relies on the application of low pressure to facilitate removal of water. The low pressure conditions decrease the boiling point of the sample and thus facilitate evaporation at low temperatures (Broeckx *et al*., 2016). This method has been used for drying of small aliquots of *Rhizobium leguminosarum* bv. *trifolii* and *Bradyrhizobium japonicum* to evaluate the effect of cultivation in crude peat extract on viability (Casteriano *et al*., 2013). Apart from that, studies on its application in drying live bacteria are limited – possibly due to the fact that lyophilization mostly confers satisfactory survival rates and has been established as a standard drying method (Broeckx *et al*., 2016) or due to the comparably long drying times of vacuum‐drying (Santivarangkna *et al*., 2007).

### Spray‐drying

Spray‐drying involves the atomization of a liquid matrix into a drying chamber with a flow of hot air, leading to quick evaporation of water, which in turn cools the sample until dry powders are formed (Morgan *et al*., 2006). Manufacturing costs are estimated to be 20% those of freeze‐drying, which makes this process more economically feasible (Santivarangkna *et al*., 2007). However, due to the quick removal of moisture and high temperatures involved, a loss of cell viability is often a major issue (John *et al*., 2011). Inlet temperatures of around 100–200°C and outlet temperatures in the range of 60–85°C are typically used when drying microorganisms (Fu and Chen, 2011). The outlet temperature seems to be one of the most critical factors for cell survival. However, it cannot be adjusted directly but is dependent on other parameters such as airflow rate, inlet temperature, liquid feed rate and solids concentration (Fu and Chen, 2011). Due to this interdependency, an elaborate fine‐tuning of the spray‐drying process is often necessary.

For robust, spore‐forming bacteria such as *Bacillus subtilis*, spray‐drying has proven applicable (Yánez‐Mendizábal *et al*., 2012). In contrast, spray‐drying more sensitive bacteria such as *Pantoea agglomerans* resulted in a decline in cell viability between two to five orders of magnitude at an outlet temperature of 90°C, depending on the carrier (Costa *et al*., 2002a,b). The survival rate can be increased by lowering the outlet temperature; however, doing so may come along with an undesirably high moisture content and clumping of the product. The authors propose using larger spray‐dryers allowing for lower outlet temperatures, thereby increasing the bacterial survival rate without negative impacts on physical product properties. Optimizing a spray‐drying protocol for cells of *Sinorhizobium meliloti*, a final cell number of around 5 × 10^9^ CFU per g (initial cell concentrations 10^11^ CFU ml^−1^) was achieved with an outlet temperature of 42°C, resulting in a final moisture content of 11% (Rouissi *et al*., 2013).

### Fluidized bed‐drying

Another prospective method is fluidized bed‐drying, which operates at temperatures of around 40°C, thus being potentially milder than spray‐drying (García, 2011). It is mostly applied as a secondary drying method to lower the residual moisture content in solid particles or granules (Broeckx *et al*., 2016). For this, the particles are suspended in an upward blowing stream of warm or hot air, conferring a fluid‐like behaviour to the bulk of the granules. Bacterial agents may either be sprayed onto this moving mass of carriers, incorporated into carriers prior to drying, e.g. as done during encapsulation in alginate or may be provided as pure dry mass and then coated with a protective shell in a fluidized bed (Broeckx *et al*., 2016). Although it is a rather low‐cost drying method, studies on fluidized bed‐drying are limited, possibly due to the infeasibility when having liquid or slurry‐like original matrices. Using a mix of vermiculite and EB^™^ (clay and wood particles) as solid carriers, a fluidized bed‐dried formulation of *P. fluorescens* was developed. During the fast drying cycle (3 h), cell viability decreased from 10^9^ to 10^5^ CFU g^−1^, but only to 10^7^ CFU g^−1^ during the slower drying cycle (20 h) (Moënne‐Loccoz *et al*., 1999).

Comparing three of the described drying methods in terms of their suitability to maintain cell viability of *P. agglomerans*, the highest reduction in CFU by four orders of magnitude was observed in spray‐drying, two in fluidized bed‐drying and one order of magnitude in freeze‐drying (Soto‐Muñoz *et al*., 2015).

### Air‐drying

Clearly, each drying method is associated with a characteristic stress regime and may therefore be more or less suitable for a given bacterial strain. It should be considered, however, that desiccation also occurs on the field under non‐controlled conditions after application of the inoculant. Evaluating a formulation regarding its viability during air‐drying is therefore highly relevant. Indeed, it has been reported that protectants working well for one drying method are not always suitable for another – as shown when developing dry formulations for *P. phytofirmans* (Berninger *et al*., 2017). In this case, gum arabic and yeast extract maintained high survival rates during freeze‐drying, but not during air‐drying, whereas the opposite was true for mannitol. It seems therefore very purposeful to investigate drying under simulated conditions matching the ones occurring on the field as closely as possible, as has been done for example in case of a *P. fluorescens* strain. Acting as an antagonist to control fire blight, its survival was investigated when sprayed onto the flowers of rosaceous plants (Bonaterra *et al*., 2007; Cabrefiga *et al*., 2011). Limited reports are available on using air‐drying for inoculum production, although it best corresponds to the drying regime occurring under natural conditions. Schisler *et al*. (2016) prepared air‐dried formulations for different *P. fluorescens* strains and obtained very good survival rates in osmoprotectants; however, drying in solid carriers resulted in a loss of cell viability by three orders of magnitude or more. Similar observations were made by Berninger *et al*. (2017) when air‐drying *P. phytofirmans* in a zeolite matrix, where survival was lower than in osmoprotectants only.

Hence, rather than only evaluating single parameters, the interplay between matrix and drying method and the resulting effect on drying kinetics, final moisture content and water activity should be taken into account. Finally, the choice of the drying method depends on the sensitivity of the strain, matrix composition and desired output form as well as practical considerations such as minimum sample size and the possibility of gnotobiotic operation. Table 2 gives an overview of the most relevant features of commonly used drying methods.

**Table 2:** Characteristics of drying methods most frequently applied during formulation development of bacterial inoculants (according to Broeckx *et al*., 2016; Fu and Chen, 2011; Santivarangkna *et al*., 2007)

	Freeze‐drying	Spray‐drying	Fluidized bed‐drying	Vacuum‐drying	Air‐drying
Minimum sample size (approximate range of laboratory scale equipment)	μl – ml	< 100 ml	< 100 ml	μl – ml	μl – ml
Typical temperature range	< 0°C	Inlet: 100–200°C Outlet: 60–85°C	30–35°C	40–70°C	25–35°C
Matrix compatibility	No limitations	Liquid matrix	Liquid matrix sprayed onto carriers; pre‐dried granular matrix	No limitations	No limitations
Output form	Cake – further processing required	Small‐sized particles	Medium‐sized particles, granules	Cake – further processing required	Cake – further processing required
Typical drying time	Hours – days	Seconds – minutes	Minutes – hours	Hours – days	Hours – days
Gnotobiotic operation	Straightforward	Challenging	Challenging	Straightforward	Challenging
Costs	Fixed: 100% Manufacturing: 100%	Fixed: 12% Manufacturing: 20%	Fixed: 9% Manufacturing: 18%	Fixed: 52% Manufacturing: 52%	Fixed: 5% Manufacturing: 18%

## Desiccation damage and protective strategies

Particularly for non‐sporulating bacteria, which do not display the ability to adopt a highly resistant, dormant form to outlast adverse environmental conditions, desiccation is a physiologically challenging process (Potts, 1994). In extremely sensitive organisms, such as the endophytic plant growth‐promoting bacterium *P. phytofirmans*, desiccation coincided with a reduction in viability by six orders of magnitude (Berninger *et al*., 2017). The damages resulting from desiccation partly depend on the drying method used, but are generally based on three main deleterious processes: oxidative damage, phase transition and browning reactions (García, 2011). In a water‐deficient system, the formation of reactive oxygen species (ROS) is a major cause for lesions of cell components. The reduced functionality of dehydrated proteins responsible for trapping such ROS and an enhanced rate of chemical processes producing ROS result in the accumulation of these free radicals (García, 2011). Subsequently, they lead to lipid peroxidation, protein denaturation and DNA mutation (Billi and Potts, 2002).

When phospholipids in the cell membrane are dehydrated, their packing density and consequently van der Waals interactions increase. The resulting rise in the phase transition temperature causes the lipids to pass from a liquid crystalline phase to a gel phase and thus to lose membrane fluidity. This renders the membrane leaky – a fact that becomes lethal particularly upon rehydration (Potts, 1994). In addition, browning reactions (Maillard reactions) cause damage derived from condensation between reducing sugars and lysine and methionine residues of proteins (Potts, 1994).

Under natural conditions, repeated desiccation is a common abiotic stress factor, occurring, for example, during drying and rewetting of soil depending on precipitation or irrigation. For non‐spore‐forming bacteria, this constitutes a potentially lethal process. To increase their desiccation tolerance, they have evolved different, complex strategies (Ramos *et al*., 2001).

**Figure 2:**
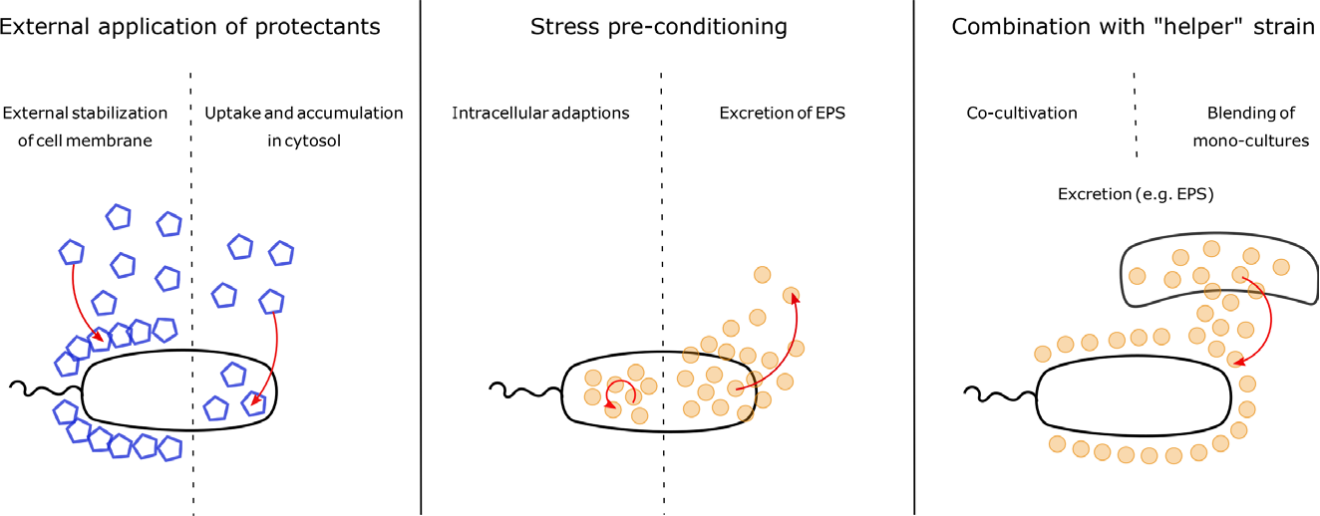
Strategies of improving desiccation tolerance in non‐sporulating bacteria. Externally added protectants stabilize the cell membrane from outside or can be accumulated in the cell (during cultivation). Stress pre‐conditioning results in intracellular adaptation (e.g. accumulation of protective agents) or secretion of EPS. Co‐cultivation with protectant‐excreting ‘helper’ strains provides external protection (e.g. by EPS).

The physiological responses of *B. japonicum*, for example, include the synthesis of the compatible solute trehalose, production of heat‐shock proteins, exopolysaccharides and enzymes for the modification and repair of DNA (Cytryn *et al*., 2007). The stress response of *Rhodococcus jostii* RHA1 involves the production of ectoine as a compatible solute as well as the synthesis of proteins protecting from oxidative stress, such as catalases (LeBlanc *et al*., 2008). The production of exopolysaccharides as a protection strategy was shown, for example, for soil *Pseudomonas* sp. (Roberson and Firestone, 1992) and *Pseudomonas putida* (Chang *et al*., 2007). Frequently, desiccation stress triggers a change in the phospholipid fatty acid profile of the cell membrane, as it was observed for example for *Pseudomonas aureofaciens* (Kieft *et al*., 1994), *Sinorhizobium meliloti*,* Bradyrhizobium elkanii, B. japonicum* (Boumahdi *et al*., 1999) and *P. putida* (Halverson and Firestone, 2000). Based on these natural protection strategies, the bacterial desiccation tolerance may be improved during the formulation process either by (i) the external addition of protectants, (ii) triggering of stress adaptation or (iii) indirect protection by a ‘helper strain’ (Fig. 2). In addition, enhancement of stress resistance by genetic engineering has been suggested (Manzanera *et al*., 2002). However, due to legal frameworks, this approach seems not viable for the time being, so it is not further discussed here.

### Externally added protectants

The most straightforward method is the external application of protectants. The list of potential protectants is extensive and comprises chemically diverse substances such as sugars, polymers and amino acids. The non‐reducing disaccharide trehalose is one of the most frequently studied desiccation protectants. The mechanism underlying its protective effect is known as the ‘water replacement hypothesis’ and is related to the ability of trehalose to lower the phase transition temperature of phospholipids in the membrane by replacing the water molecules around the lipid head groups (Leslie *et al*., 1995). This helps maintaining the membrane fluidity and thus integrity. Trehalose or other non‐reducing sugars may also form hydrogen bonds with proteins when water is absent, thereby preventing protein denaturation during desiccation (García, 2011). Furthermore, vitrification, that is the glass formation of trehalose and other sugars, is assumed to aid in the protection of cells by stabilizing the cytoplasm (Potts, 1994). Despite the good protective effect of trehalose, it may be necessary to consider more economic solutions. Alternative protectants – including those used in bacterial formulations for food technology – are skimmed milk, liquid growth medium, horse serum (Peiren *et al*., 2015), sucrose, Ficoll, hydroxyethylcellulose, hydroxypropylmethylcellulose, polyvinylalcohol (Wessman *et al*., 2011), glucose, sucrose, maltodextrin (Strasser *et al*., 2009), fructose, lactose, sodium glutamate, cysteine, dextran, polyethyleneglycol and glycerol (Costa *et al*., 2000). Table 3 gives an overview of protectants applied in formulation of agriculturally relevant strains. In many cases, mono‐ and disaccharides proved most efficient. For example, different *Pseudomonas* strains were best stabilized by adding 20 g per litre fructose or trehalose (Schisler *et al*., 2016) or lactose (Cabrefiga *et al*., 2011), whereas *P. agglomerans* was best protected by adding sucrose (Costa *et al*., 2000).

**Table 3:** Studies on the improvement of survival of non‐sporulating, agriculturally relevant bacteria in different drying methods, considering protectants and additional relevant parameters

Bacterial agent	Protectants	Other parameters	Drying	Parameters supporting viability in dry state	References
*Azospirillum brasilense*		Encapsulation in alginate, skimmed milk for release	AD FD	FD	(Bashan *et al*., 2002)
*Azospirillum brasilense*		Cell flocculation	AD	Cell flocculation	(Joe *et al*., 2012)
*Azospirillum brasilense, Raoultella terrigena*	Trehalose	Encapsulation in alginate, filler, gelling agent, growth stage at harvesting	AD	Stationary phase Gelling agent CaCl_2_ for *A. brasilense*, calcium gluconate for *R. terrigena* Starch as filler Trehalose as protectant	(Schoebitz *et al*., 2012)
*Azospirillum lipoferum*	Skimmed milk, starch, humic acid	Encapsulation in alginate	AD	0.8% humic acid as protectant	(Reetha *et al*., 2014)
*Bradyrhizobium japonicum*	Skimmed milk‐sucrose	Shelf life (relative humidity, temperature)	SD	Storage at 4°C and low % relative humidity under vacuum or nitrogen atmosphere	(Mary *et al*., 1993)
*Bradyrhizobium japonicum*	Trehalose	Trehalose supply during growth vs. after harvesting	AD	Trehalose supply during growth	(Streeter, 2003)
*Enterobacter* sp., *Serratia sp*., *Microbacterium* sp., *Pseudomonas* sp., *Achromobacter* sp.	Skimmed milk	Consortia formulation	FD	Consortium with *Pseudomonas* sp.	(Barra *et al*., 2016)
Fluorescent *Pseudomonas*		Glycerol as carbon source, CMC as adhesive, inorganic carriers	AD	Vermiculite as carrier	(Sarma *et al*., 2011)
*Lysobacter capsici*	Arabic gum, chitosan, CMC, corn steep liquor, gelatin, glycerol, molasses, paraffin, pinolene, polyacrylate, PEG, PVA, PVP, skimmed milk, alginate, sorbitol, starch, xanthan	Growth conditions, shelf life, UV protection, wash‐off	AD	PEG as desiccation protectant	(Segarra *et al*., 2015)
*Methylobacterium oryzae, Methylobacterium suomiense, Azospirillum brasilense*		Encapsulation in alginate, co‐aggregation	AD	Co‐aggregation with *Methylobacterium oryzae*	(Joe *et al*., 2014)
*Pantoea agglomerans*	Trehalose, glucose, fructose, sucrose, lactose, sodium glutamate, cytine, dextran, PEG, glycerol, NFSM	Cell load, rehydration media	FD	High initial cell load, sucrose as protectant, rehydration in NFSM	(Costa *et al*., 2000)
*Pantoea agglomerans*	MgSO_4_, NFSM, MgSO_4_‐NFSM	Rehydration media	SD	MgSO4 or MgSO4‐NFSM as protectants Rehydration in NFSM	(Costa *et al*., 2002a)
*Pantoea agglomerans*	Sucrose (different concentrations)	Cell load, rehydration media, storage conditions	FD	Rehydration in 1% NFSM, 10% sucrose as protectant, storage at 4°C in high barrier plastic bags under vacuum or in glass vials	(Costa *et al*., 2002b)
*Pantoea agglomerans*	MgSO_4_ (in SD), sucrose (in FD), starch (FBD)	Rehydration media	FD SD FBD	FD, rehydration in NFSM	(Soto‐Muñoz *et al*., 2015)
*Paraburkholderia phytofirmans*	Trehalose, sucrose, galactose, lactose, sorbitol, mannitol, glycerol, CMC, gelatin, arabic gum, Ficoll, humic acid, maize starch, maltodextrin, skimmed milk, DMSO, yeast extract, EPS	Storage temperature, inorganic carrier	AD FD	Air‐drying, skimmed milk as protectant, storage at 4°C	(Berninger *et al*., 2017)
*Pseudomonas aeruginosa*	EPS	Inorganic carrier	AD	EPS as protectant	(Tewari and Arora, 2014a,b)
*Pseudomonas fluorescens*	Lactose	Growth conditions (media, time, temperature, heat shock, pH change)	FD	Harvesting after 16 h, incubation at 25 or 30°C, mild heat shock at 35°C	(Bisutti *et al*., 2015)
*Pseudomonas fluorescens*	Glycine betaine	Osmoadaptation by NaCl addition to growth media	VD	Osmoadaptation (0.7 M NaCl)	(Bonaterra *et al*., 2007)
*Pseudomonas fluorescens*	Glycine betaine	Osmoadaptation, nutrient addition	AD	Osmoadaptation (0.7 M NaCl and 0.1 mM glycine betaine), addition of 50 mM glycine and 5 mM Tween 80	(Cabrefiga *et al*., 2011)
*Pseudomonas fluorescens*	Lactose, skimmed milk, sucrose, starch, trehalose, lactose‐starch, skimmed milk‐starch, trehalose‐starch	Osmoadaptation	FD	Osmoadaptation Lactose as protectant Storage at 4°C	(Cabrefiga *et al*., 2014)
*Pseudomonas fluorescens*	Lactose	Mineral carriers, relative humidity at storage	AD	Mineral with low surface area supplemented with lactose as protectant	(Dandurand *et al*., 1994)
*Pseudomonas fluorescens*		Accelerated storage test	FD	Storage at 4°C	(Jean‐Noël *et al*., 2012)
*Pseudomonas fluorescens*		Incubation procedure, nutrient amendment	FBD	Incubation on carrier, sucrose asparagine broth as amendment, slow drying	(Moënne‐Loccoz *et al*., 1999)
*Pseudomonas fluorescens*	Glucose, fructose, trehalose, raffinose, stachyose	Inorganic carriers	AD	20 g per l trehalose or fructose as protectants, Kenite^®^ 700 or HYFLO^®^ as carriers	(Schisler *et al*., 2016)
*Pseudomonas fluorescens*	EPS (marginalan)		AD	EPS as protectant Storage at 4°C	(Slininger *et al*., 2010a,b)
*Pseudomonas fluorescens, Enterobacter cloacae*	For *E. cloacae*: lactose + bovine serum albumen, lactose, sucrose, fructose); for *P. fluorescens*: buffer, spent broth, sucrose, stachyose, raffinose, melezitose, trehalose, lactose	Growth media, growth stage, storage atmosphere and temperature	AD	Culture age 24–48 h if in buffer, 72–96 h if in spent broth; generally stachyose best protectant	(Slininger and Schisler, 2013)
*Pseudomonas fluorescens‐putida*	Hydrophilic silica (Sipernat^®^)	Encapsulation in Eudragit^®^, storage relative humidity	SD	Silica as protectant when stored at low relative humidity	(Amiet‐Charpentier *et al*., 1999)
*Pseudomonas putida*	Trehalose‐PVP, hydroxyectoine‐PVP	Osmoadaptation	VD	Osmoadaptation by 0.4 M NaCl in growth media, 1 M hydroxyectoine as protectant	(Manzanera *et al*., 2002)
*Pseudomonas putida, Pseudomonas fluorescens, Pseudomonas chlororaphis*	Saccharose, lactose, lignosulfonic acid, glucose, skimmed milk, starch, CMC, nutrient broth, egg white albumen, egg yolk, lecithin, SPAN 60‐xanthan, SPAN 80‐xanthan, Na‐alginate, xanthan, Na‐glutamate, glycerol, bentonite, activated carbon, alkalic lignin	FD parameters (freezing rate, shelf temperature)	FD	Saccharose, lactose, skimmed milk as protectant; shelf temperature 20 –30°C	(Stephan *et al*., 2016)
*Pseudomonas putida, Sphingomonas* sp.	Sucrose, Ficoll, HEC, HPMC, PVA		FD	Sucrose and Ficoll as protectants	(Wessman *et al*., 2011)
*Pseudomonas trivialis*	Sucrose and/or corn oil	Mineral carriers Storage temperature	AD	Sucrose‐corn oil as protectant, Pesta as carrier, storage at 4°C	(Mejri *et al*., 2013 )
*Rhizobium leguminosarum* bv*. trifolii*	PVA, PVA‐canola oil, polyvinyl acetate latex		AD	Storage at 0.38–0.47 water activity, PVA‐canola oil as protectant at low water activity	(Deaker *et al*., 2012)
*Rhizobium leguminusarum* bv. *trifolii,Bradyrhizobium japonicum*		Growth media	VD	Crude peat extract as culture media	(Casteriano *et al*., 2013)
*Rhizobium tropici, Ryhizobium etli*	Trehalose, sucrose‐peptone	Storage temperature	FD	Trehalose as protectant, storage at 4°C	(Pereira *et al*., 2002)
*Sinorhizobium meliloti*	Cheese whey powder + sucrose + sorbitol	SD parameters (inlet temperature, drying rate, spray pressure, feed sample rate)	SD	Inlet temperature 105°C, air‐drying rate 0.56 m^3^ per min, spray pressure 0.07 MPa, feed flow rate 8 ml per min	(Rouissi *et al*., 2013)
*Sinorhizobium meliloti, Bradyrhizobium elkanii, Bradyrhizobium japonicum*		Growth stage, storage relative humidity	AD	Long‐term storage: lag phase for *S. meliloti* and *B. elkanii*, stationary phase for *B. japonicum*; 44% for *B. elkanii*, 44–68% for *B. japonicum*, 22–44% for *S. meliloti*	(Boumahdi *et al*., 1999)

EPS, exopolysaccharide; CMC, carboxymethylcellulose; DMSO, dimethyl sulfoxide; PEG, polyethylene glycol; PVA, polyvinylalcohol; PVP, polyvinylpyrrolidone; RH, residual humidity; NFSM, non‐fat skimmed milk; FD, freeze‐drying; AD, air‐drying; SP, spray‐drying; VD, vacuum‐drying; FBD, fluidized bed‐drying.

Commonly, the protectants are added externally to the bacterial cells after harvesting from the broth and prior to drying. However, it has also proven feasible to add trehalose to the culture medium and achieve a protective effect through its uptake and accumulation in the cytoplasm. For example, in the case of *B. japonicum*, trehalose was accumulated in cells during growth and thus had a better protective effect than when added after growth (Streeter, 2003). On the other hand, Schoebitz *et al*. (2012) found that amending the culture medium with trehalose improves the survival during subsequent drying of *Raoultella terrigena*, but not of *Azospirillum brasilense*. Thus, this strategy is not applicable for all strains, especially if trehalose is metabolized during growth rather than accumulated in the cell (Streeter, 2003).

Furthermore, the osmotic balance seems to be an important aspect during the addition of protectants to bacterial cultures (Wessman *et al*., 2011). When using osmotically active substances such as mono‐ and disaccharides, iso‐osmotic conditions should be maintained. Therefore, the concentration of the protectant needs to be adjusted to reach a similar osmolarity as the growth medium. This reduces the osmotic shock upon transfer from growth medium to drying matrix, which may occur too fast for bacteria to physiologically adapt. When applying high‐molecular weight polymers as protectants (e.g. Ficoll or hydroxyethylcellulose), salts such as NaCl might be added to reach the desired osmolarity. To give an example, a 10% (w/v) solution of sucrose in dH_2_O has an osmolarity of 0.34 Osmol per litre. In order to achieve iso‐osmotic conditions, high‐molecular weight polymers need to be amended with approximately 150 mM of NaCl. Maintaining iso‐osmotic conditions also facilitates the assessment of various protective agents applied in equal concentrations, which are otherwise difficult to compare due to differences in osmotic pressure.

An interesting approach to isolate new, potential desiccation protectants – termed ‘xeroprotectants’ – was pursued by Narváez‐Reinaldo *et al*. (2010). The authors isolated non‐sporulating bacteria from soil subjected to seasonal drought, and triggered the accumulation of their characteristic xeroprotectants by subjecting them to slow air‐drying. Subsequently, the cells were resuspended in deionized water to release their xeroprotectants due to the osmotic gradient (‘dry milking’). A compositional analysis by NMR showed strain‐specific ratios of a mix of protective substances, comprising fructose, glutamic acid, acetate, β‐hydroxybutyrate, lactate, glucose, valine, trehalose, oxoglucuronic acid, glutamine, fucose and pyruvate. Thus, it seems that desiccation tolerance may best be conferred by synergistically acting compounds. When artificially synthesizing the mixtures, some of them proved suitable to stabilize *Escherichia coli* during drying. This method may give valuable insights into the characteristic composition of strain‐specific xeroprotectants to more precisely match the composition and concentration of externally applied protectants.

### Stress adaptation

As an alternative to the external application of protectants, cellular protective mechanisms may be triggered by applying sublethal stress prior to desiccation. This provokes the modulation of cell physiology to adapt to the perceived environmental stress and thereby indirectly enhances desiccation tolerance.

For example, fermentation under suboptimal pH or temperature conditions was found to influence the composition of membrane lipids of lactobacilli insofar as it increased the ratio of unsaturated to saturated fatty acids. The lower phase transition temperature exhibited by unsaturated fatty acids rendered the cell membrane less prone to damage when drying (Schoug *et al*., 2008; Liu *et al*., 2014). Growing the bacterial culture to stationary phase may also induce certain stress responses due to the depletion of nutrients and accumulation of toxic metabolites (Morgan *et al*., 2006). These adverse conditions may, similar to salt stress or suboptimal temperature or pH, aid in preparing bacteria for the following desiccation stress, for example by synthesizing different stress proteins. However, it has been observed that the correlation between growth phase and desiccation tolerance is also strain‐dependent (Schoebitz *et al*., 2012).

For example, *P. fluorescens* was shown to intracellularly accumulate the osmolytes trehalose, *N*‐acetylglutaminylglutamine and glucosyl‐glycerol when subjected to hyperosmotic stress by adding 0.7 M of NaCl to the growth medium (Bonaterra *et al*., 2007). This procedure substantially increased the survival after spraying on apple plants. Similar results were obtained by Cabrefiga *et al*. (2011), who showed that osmoadapted *P. fluorescens* had an enhanced survival rate after inoculation on aerial plant parts, especially at low relative humidity. The same strategy enhanced the survival of *P. fluorescens* during freeze‐drying with lactose from 70% to 100% (Cabrefiga *et al*., 2014). Furthermore, *Rhizobium etli* osmotically pre‐conditioned by adding 0.2 M NaCl to the growth medium showed survival rates of 35% after vacuum‐drying as opposed to 0.01% without pre‐conditioning (Reina‐Bueno *et al*., 2012).

Improvement in desiccation tolerance was also achieved by applying mild heat shocks of 35°C for 1 h to *P. fluorescens*. This increased the survival after freeze‐drying by almost 80%, which has been ascribed to the synthesis of heat‐shock proteins in response to increased temperatures (Bisutti *et al*., 2015). In contrast, acid adaptation by fermentation under suboptimal pH did not improve survival.

In a study involving rhizobia, the presence of an aqueous peat extract in the growth medium was found to trigger adaptive responses. These included the enhanced expression of proteins for protection and repair of cell components as well as trehalose accumulation. The subsequent effect on desiccation tolerance differed depending on the strain: *Rhizobium leguminosarum* bv. *trifolii* showed an 18 times higher survival rate, whereas the tolerance to drying was not significantly improved in *B. japonicum* (Casteriano *et al*., 2013).

Instead of adapting a given strain to stress conditions, another practical approach may be the selection of strains that are intrinsically tolerant to desiccation. For example, populations of *R. leguminosarum* bv. *trifolii* isolated from dry soil environments showed a greater survival after rapid drying than those isolated from moisture saturated soils (van Ham *et al*., 2016).

The idea of using selective pressure to obtain more robust phenotypes may be simulated under laboratory conditions using adaptive evolution (Dragosits and Mattanovich, 2013). Due to their short generation cycle, large populations and asexual reproduction, bacteria are ideal organisms to study genomic evolution within a relatively short time frame. Under certain stress conditions, spontaneously occurring mutations lead to an increased fitness of individual cells. Ultimately, the variants with the acquired beneficial traits gradually replace the original population. To date, this principle has been applied to industrially relevant *E. coli* rather than to plant‐associated bacteria. For example, *E. coli* populations subjected to repeated cycles of freezing, thawing and growing were improved regarding their fitness by up to 90% over the course of 1000 generations (Sleight and Lenski, 2007). Similarly, Dragosits *et al*. (2013) adapted *E. coli* to different stressors such as acidity and osmotic pressure. In many cases, the acquired resistance was due to single nucleotide polymorphism. In pursuing adaptive evolution, it has to be considered that genome alterations may also result in undesirable properties. However, when such a trade‐off can be excluded, adaptive evolution provides an interesting, underused tool for the improvement of desiccation tolerance in inoculant strains.

### Exopolysaccharides

The secretion of exopolysaccharides (EPS) is a bacterial defence mechanism against environmental stressors such as drought, predation, competition and toxic compounds (Patel and Prajapati, 2013). For example, soil inhabiting *Pseudomonas* sp. was found to produce EPS in response to drying stress and thereby create a microenvironment with increased water retention capacity and reduced drying rate (Roberson and Firestone, 1992). Indeed, including EPS as a matrix component in formulations has been recognized as a novel strategy to protect cells and support plant colonization (Arora and Mishra, 2016). One of the earliest reports describing this approach concerns the improvement of the shelf life of an inoculant based on *Klebsiella oxytoca* and *Xanthomonas maltophilia*, using the EPS mucilan produced by *Bacillus mucilaginosus* (Kozyrovska *et al*., 1997). More recently, the relevance of EPS was shown by Tewari and Arora (2014b), who triggered EPS production of *Pseudomonas aeruginosa* by cultivating the bacteria in medium amended with up to 1.6 M NaCl. Then, the bacterial suspensions were mixed with talc as a carrier and applied as a coating to sunflower seeds. The EPS‐containing formulations did not only show a higher shelf life, but also resulted in a 50% increased germination rate of sunflower under saline conditions as compared to inoculation with an EPS‐deficient mutant. Furthermore, root length, shoot length, head diameter and yield were significantly increased due to inoculation with *P. aeruginosa* in the EPS‐containing formulation. Moreover, the biocontrol activity of the same strain against the sunflower pathogen *Macrophomia phaseolina* was demonstrated, again proving the superior performance of the inoculant strain in the presence of EPS under saline conditions (Tewari and Arora, 2014a). This demonstrates the tremendous influence of EPS not only on cell viability, but also on plant colonization, growth promotion and biocontrol activity.

In addition, the importance of the EPS marginalan for the performance of *P. fluorescens* was demonstrated (Slininger *et al*., 2010a). Depending on drying method and storage conditions, the viability was maintained up to five orders of magnitude higher in the presence of marginalan. Interestingly, this EPS did not only have a protective effect on the cells producing it, but also on other, non‐EPS producing strains of *P. fluorescens*, albeit not to the same extent. Another study showed the potential benefit of retaining *P. fluorescens* in the growth medium rather than washing the cells and thus removing EPS (Slininger and Schisler, 2013). Omitting the washing resulted in the cells being less susceptible to drying.

Exopolysaccharides is also known to be produced by *Burkholderia* and *Paraburkholderia* strains and is triggered in the presence of certain carbon sources during cultivation, for example sugar alcohols such as mannitol and glucitol (Bartholdson *et al*., 2008). The most common EPS type among these strains is termed cepacian and consists of a branched acetylated heptasaccharide repeat unit with D‐glucose, D‐rhamnose, D‐mannose, D‐galactose and D‐glucuronic acid (Cérantola *et al*., 2000; Cescutti *et al*., 2000). It was shown to provide protection against desiccation and metal ion stress in several environmental strains (Ferreira *et al*., 2010), which makes it a prospective protectant in formulation development. In fact, EPS improved viability of *P. phytofirmans* during air‐drying by up to six orders of magnitude compared with the control (Berninger *et al*., 2017).

Similar to being embedded in EPS networks, the aggregation of bacterial cells in bioflocs seems to confer some resistance to environmental stresses. For example, *Azotobacter* and *Paenibacillus* were better able to withstand environmental stresses such as heat, desiccation and elevated salt levels when contained in bioflocs (Kalaiarasi and Dinakar, 2015).

Overall, the use of EPS as a component of microbial formulations has a high potential to increase viability and performance of microbial inoculants.

### Formulation in consortia

Protective, secondary metabolites such as EPS might not only benefit the producing strain itself, but may simultaneously confer protection to other (e.g. co‐formulated) bacteria. To date, research regarding appropriate formulations of microbial consortia has mainly been focused on the effects on the plant itself, such as synergistic mechanisms of partner strains resulting in better biocontrol or growth promotion. For example, biofilmed inoculants were developed based on cyanobacteria or the fungus *Trichoderma* as matrix formers and legume inoculants (e.g. *B. japonicum* and *P. fluorescens*) as partners (Prasanna *et al*., 2013). This led to increased yields of soya bean and mung bean, which the authors attributed to the improved establishment of bacteria in the rhizosphere due to the provision of nutrients and protection in the biofilm matrix. However, the influence of secondary metabolites of the ‘helper’ strain on the viability of sensitive, co‐formulated partners often remains unclear.

Furthermore, the possibility of co‐cultivation of microorganisms with different functions was evaluated. For example, an EPS‐providing *Paenibacillus* sp. ‘helper’ strain was co‐cultivated with Gram‐negative biocontrol and biofertilizer strains (*K. oxytoca, Pseudomonas sp*., *P. fluorescens, P. aureofaciens, P. agglomerans, Agrobacterium* sp.) (Kozyrovska *et al*., 2005). These combinations proved to be compatible for co‐cultivation, i.e. no impairment in their growth was observed. After 60 days in dual culture, the *Pseudomonas* sp. strain showed a 100 times higher viability than when grown in monocultures. No drying step was included, nevertheless the results indicate that the EPS produced by *Paenibacillus* sp. had a protective effect on the Gram‐negative strains and might also serve as a carbon source. The approach of co‐cultivation instead of a two‐stage process (growth of partner strains in monocultures followed by mixing) is economically more feasible, but the strains have to be compatible. Co‐cultivation versus blending was investigated for *P. fluorescens* and *Enterobacter cloacae* strains (Slininger *et al*., 2010b) *. *When grown together, certain strain combinations suppressed dry rot in potato significantly better than when blended after monocultivation. This effect was partly ascribed to the community benefit provided by EPS producing *P. fluorescens* strains.

Choosing potential partners for co‐formulation is facilitated when knowledge about their extracellular compounds is available. An innovative approach to gain this insight is the analysis of the exoproteome, as it was performed by Lidbury *et al*. (2016) to investigate the physiological adaptations of *Pseudomonas* strains in a phosphorus‐depleted soil. An enhanced secretion of certain enzymes related to phosphate uptake was shown to be triggered by low phosphate conditions. A subsequent study proved that even co‐cultured bacteria could benefit from this extracellularly occurring metabolism (Lidbury *et al*., 2017). Although this example is related to the nutrient provision of co‐formulated partners, it might be transferred to the provision of desiccation protectants. Possibly, exoproteomic studies help in elucidating suitable culture conditions to support the secretion of protective compounds.

## Methods for viability assessment

### Cell viability during formulation

Reliable, high‐throughput and cost‐efficient methods for the determination of bacterial viability are needed to facilitate the screening of a large range of processing parameters. Important features of a given method are the detection limit and dynamic range as well as the suitability to investigate different types of matrices, including solid and/or opaque ones (Braissant *et al*., 2015). This is relevant when checking the viability of immobilized or encapsulated bacteria or when using e.g. skimmed milk as protectant or rehydration media. Table 4 provides an overview of the methods described in this section.

**Table 4:** Characteristic features of some methods for determination of cell viability in bacterial inoculants

	Plate‐counting^a^	Plate reader – absorbance (OD_600_)^a^	Plate reader – luminescence (BacTiter‐Glo^™^) b	Plate reader – fluorescence (LIVE/DEAD *Bac*Light^™^) b	Microscopy – fluorescence (LIVE/DEAD *Bac*Light^™^)^b^	Flow cytometry – fluorescence (LIVE/DEAD BacLight^™^)^b^	PMA‐qPCR^a^	Microcalorimetry^a^	Intracellular phototautomerismc
Approximate detection limit	1 CFU (resolution depending on incubated volume)	10^7^ cells per ml (lower when measuring growth time to detection)	10^1^ to 10^3^ cells per well (100 μl), depending on bacterial species	10^5^ to 10^6^ cells per ml, depending on bacterial species	2 × 10^5^ to 2 × 10^6^ cells per ml (depending on bacterial species)	10^4^ cells per ml	10^2^ cells (depending on dead cell background)	3 × 10^4^ cells per ml	10^6^ cells per ml
Duration of assay	Days	Minutes	Minutes	Minutes	Minutes to hours	Minutes to hours	Hours	1 Hour	Minutes to hours
Opaque samples	Possible	Limited (depending on ratio of sample to rehydration media)	No	No	No	No	Limited	Possible	No
Specificity	Limited (selective medium, morphology)	No	No	No	No	No	High	No	No
Automation possible	Limited	Yes	Yes	Yes	No	Yes	Yes	Yes	Yes
Decisive parameter for detection	Culturability	Cell biomass	Metabolic activity (ATP)	Membrane integrity	Membrane integrity	Membrane integrity	Membrane integrity	Metabolic activity (heat)	Cytosolic pH homeostasis

**a.** According to Braissant *et al*. (2015) and Davis (2014).

**b.** According to the manufacturer's instructions (LIVE/DEAD^®^ BacLight™ Bacterial Viability Kit, Thermo Fisher Scientific Waltham, MA, USA; BacTiter‐Glo™ Microbial Cell Viability Assay, Promega, Fitchburg, WI, USA).

**c.** According to Kort *et al*., 2010.

Cultivation on solid media followed by counting the colony‐forming units has been the gold standard for enumeration of live bacteria. It provides the highest dynamic range, theoretically allowing for detection of a single cell. However, it is laborious, time‐consuming and delivers results only after 24–48 h of incubation – in some cases of slow growing bacteria even more. As contaminants interfere with plate‐counting, this method is suitable only when working under gnotobiotic conditions throughout the formulation tests. A limited specificity can be achieved if employing selective growth media for the strain of interest. For *B. japonicum* for example, the suppression of most fungi and Gram‐positive bacteria was achieved by adding pentachloronitrobenzene and vancomycin to the yeast–mannitol medium, thus making plate counts more specific (Penna *et al*., 2011). Culturing on solid medium may be performed in a higher throughput when preparing samples in multiwell plates, using multichannel pipettes and incubating droplets of small volumes (5–10 μl) instead of streaking the sample (Herigstad *et al*., 2001; Nocker *et al*., 2012). However, reducing the incubation volume results in a lower resolution.

Cell numbers have also been monitored by absorbance spectrophotometry, determining the optical density at a specific wavelength and correlating it to the amount of bacterial cells present in the sample. Optical density does not discriminate between dead and live cells and has a higher detection threshold of about 10^7^ cells per ml (Braissant *et al*., 2015). However, taking into account the time of incubation until this threshold is reached, a considerably lower number of live cells may be detected. Assuming that the regrowth time after rehydration is proportional to the number of surviving cells, Hazan *et al*. (2012) reported the detection of as few as 10 cells per ml. In addition, the authors achieved a good resolution which allowed them to distinguish between 40 and 400 cells per ml in the original sample. A similar approach was pursued by Slininger and Schisler (2013), who dried microdroplets (1 μl) of bacterial suspensions in different protectants and subsequently rehydrated them in excess growth media. Monitoring the growth kinetics, they rapidly identified suitable formulation parameters for eight strains simultaneously. Using a high ratio between the rehydration volume and original sample volume has the advantage that cell debris or components of the formulation (e.g. protectants or carriers) become negligible in terms of their effect on absorbance. This implies a limited compatibility with solid and opaque samples. Nevertheless, this method requires thorough calibration for each individual strain, to account for strain‐specific variations in growth kinetics.

Another aspect to consider is that cultivation‐based methods do not detect those cells, which may have acquired a so called viable but non‐culturable (VBNC) state due to stress during technological processing. These cells are structurally intact and may be resuscitated under appropriate circumstances (Basaglia *et al*., 2007). Assessment of survival rates is therefore also a question of defining bacterial viability, which may be based either on culturability or on structural integrity.

Structural integrity is often monitored using a combination of fluorescent dyes, one of which selectively stains nucleic acids of cells with compromised membranes, whereas the other dye labels all cells. Typically, green fluorescent SYTO 9 is combined with red fluorescent propidium iodide as a selective dye. The ratio of red to green fluorescent signal may then be determined by microscopy or, allowing for higher throughput, by fluorescent spectroscopy in a plate reader. Applying this method to enumerate *Sinorhizobium meliloti* proved the presence of a fraction of intact but non‐culturable cells after drying and storage and provided information about the physiological state of cells (Vriezen *et al*., 2012). Similar observations were made by Nocker *et al*. (2012), who investigated the response of *E. coli*,* P. aeruginosa*,* Enterococcus hirae* and *Staphylococcus aureus* to air‐drying in a multiparameter assay. This assay relied on live/dead staining and culturing on solid media on the one hand, and functional parameters such as membrane potential, esterase and respiratory activity on the other hand. Although the authors could gain valuable insight into stress levels at a given point of measurement, the cultivation‐independent methods could not fully replace standard plate‐counting. This was because of the limited dynamic signal range detectable by the plate reader. The signal frequently fell below the detection threshold, especially when dealing with severely stressed and sensitive bacteria.

Live/dead staining has also been suggested as an efficient and time‐saving detection method to monitor viability of lactic acid bacteria starter cultures in food industry by help of flow cytometry (Kramer *et al*., 2009; Díaz *et al*., 2010). Flow cytometry measures viability at the single cell level by creating optical signals from scattering or fluorescence when the cells pass through a laser beam. The resulting signal may not only be correlated to membrane integrity using fluorescent dyes, but also to other structural and functional parameters. This method is not commonly applied in development of plant inoculants, but rather in pharmaceutical and food industry as well as for monitoring pathogens. To give one example, Bensch *et al*. (2014) reported the monitoring of viability of lactobacilli after fluidized bed‐drying by flow cytometry, in which fractions of viable, damaged and dead cells could be differentiated.

Viability assays may also be based on biochemical reactions with certain cell components, e.g. sugars or amino acids, on metabolic activity or on the turnover of chromogenic or fluorogenic substrates by cellular enzymes (Braissant *et al*., 2015). Biochemical assays can be suitable for estimating the viability in encapsulated formulations as they allow for *in situ* measurements under certain circumstances. This is an advantage over standard plate‐counting, which requires dissolution of the encapsulation matrix to release the cells. In an approach targeting bioreducible tetrazolium salt and adenosine triphosphate, the viability of *E. coli* encapsulated in alginate and polyvinylalcohol was investigated and could reliably be determined in case of the alginate matrix (Wadhawan *et al*., 2010).

In another innovative approach, viable cells, e.g., of *Bacillus subtilis* were detected by intracellular phototautomerism (Kort *et al*., 2010). This method relies on the pH difference in the cytosol of live and damaged cells. A specific, neutral probe, which can penetrate the cell membrane, dissociates into a fluorescent phototautomeric anion only at the neutral pH in live cells. The emitted signal was monitored by help of a microplate reader at a detection limit of 10^6^ CFU ml^−1^.

As an alternative, DNA amplification methods may be applied to specifically detect the strain of interest. However, DNA may persist even if the bacterial cell is already compromised and thus does not allow for discrimination between live and dead cells. To achieve this, samples may be incubated with ethidium monoazide (EMA) or propidium monoazide (PMA). These DNA‐intercalating dyes are only able to penetrate the cells through permeabilized membranes (Nocker and Camper, 2009). Subsequent to incubation with one of these dyes, the samples are exposed to bright light to induce the covalent binding of the dye to DNA. As a result, amplification of DNA is inhibited during PCR, so only DNA from cells with intact membranes that prevented the penetration of EMA or PMA is detected. Combining this approach with real‐time quantitative polymerase chain reaction (qPCR) allows for the estimation of the number of bacterial cells with an intact membrane. However, this promising approach still needs to be evaluated for different bacterial strains and stress regimes, as these are relevant parameters for the conditions of cell membranes and the ability of the dyes to enter (Nocker and Camper, 2009; Løvdal *et al*., 2011). The exclusion of false positives requires thorough optimization of the protocol, especially when dealing with a high background microflora (Gensberger *et al*., 2014). Frequently, there seem to be issues with false‐positive results, which may arise from an inefficient light activation especially in turbid samples or in high initial cell concentrations (Løvdal *et al*., 2011).

In most cases, viability of agriculturally relevant inoculants is assessed by standard plate‐counting. Other approaches, such as the use of DNA‐based methodologies (specifically PMA‐qPCR) have been tested for enumeration of *P. agglomerans* in a biocontrol product (Soto‐Muñoz *et al*., 2015). Comparing the results to numbers obtained by plate‐counting, the authors found a good agreement between both methods when bacteria were freeze‐dried or fluidized bed‐dried. After spray‐drying, however, cell numbers obtained by PMA‐qPCR (assessing intact cells) were two orders of magnitude higher those obtained by plate‐counting. This indicates that a loss of culturability does not necessarily coincide with membrane damage. Therefore, the feasibility of PMA‐qPCR seems to depend on the drying method, as the membrane perforation and thus the ability of PMA to enter the cells is influenced by the type of stress regime experienced.

Another possible method to measure cell growth and activity is isothermal microcalorimetry (reviewed by Braissant *et al*., 2015). Generally, isothermal microcalorimetry relies on the detection of heat released as a result of physiological or chemical processes as they occur in metabolically active cells. Its low detection limit of approximately 10^4^ cells and the possibility to use it in solid and opaque samples make it a promising, innovative tool for quality control in formulations. In fact, this method has successfully been used for viability assessment in lactobacilli applied in probiotic industry (Garcia *et al*., 2017), enabling the detection of 3 × 10^3^ CFU ml^−1^ within 10 h. Although analysis of data obtained by this method may require some training, the improvement in handling and capacity of new instruments is likely to support a wider application of this technology in the future (Braissant *et al*., 2015).

The decision on which method to apply for evaluating viability of bacterial inoculants thus depends on the composition of the formulation (opaque, solid), available equipment and expertise, expected number of live and dead cells, definition of viability as well as access to strain‐specific information. The latter facilitates the application of methods, which otherwise require labour‐intensive design and evaluation, such as PMA‐qPCR. Generally, a two‐step approach based on an initial, low‐precision method in combination with a subsequent, high‐resolution method may be preferable. For example, the time to regrowth after rehydration can be measured in high throughput in a plate reader or colony‐forming units can be determined by drop‐plating. In the second step, unfavourable strain formulation combinations are dismissed, and a selection of the most promising ones is further examined by high‐resolution methods. These could be based on live/dead staining, which gives additional information about the physiological and structural characteristics of the cells. Such a two‐step approach may be the best way to balance economic constraints and large‐scale screening of formulation parameters.

### Cell viability throughout storage

A sufficiently long shelf life is a prerequisite for successful commercialization of a microbial biocontrol or biofertilizer product. Requirements regarding shelf life vary, ranging from 2 to 3 months at room temperature (Malusá *et al*., 2012) over 1–1.5 years (Catroux *et al*., 2001) to a minimum of 1–2 years (Bashan *et al*., 2014). Maximizing the initial amount of cells in the inoculant to compensate for a fast rate of deterioration is not economically efficient. Instead, storage conditions should be optimized to support long‐term cell survival. Factors detrimental to storage stability include exposure to oxygen, high temperature, moisture, microbial contamination and light (Morgan *et al*., 2006). Investigating the effect of packaging, it was found that high barrier plastic bags or glass vials were more suitable for maintaining cell viability of *P. agglomerans* than low barrier plastic bags, presumably due to the exclusion of oxygen and moisture (Costa *et al*., 2002b).

Apart from temperature, water activity, which describes the availability of water in a sample, has been recognized as a critical parameter influencing storage stability. Highest survival rates of rhizobia coated onto seeds were achieved with water activities between 0.47 and 0.38. However, survival also depends on the seed species, inoculum preparation, coating ingredients and coating technique (Deaker *et al*., 2012). Thus, while there are some generally valid strategies to enhance shelf life, the fine‐tuning of storage conditions regarding the aforementioned parameters seems to be strain‐specific as is the case for the desiccation process.

As the evaluation of storage stability over prolonged periods slows down the process of formulation optimization, accelerated shelf life tests may be performed. Modelling the loss of viability over time is one approach and is mostly based on the Arrhenius model, assuming a temperature‐dependent deterioration rate. Viability data acquired from short‐term storage at elevated temperatures can thus be used to predict the survival rate at lower temperatures for any period of time. Limited reports are available regarding agriculturally relevant strains, such as *P. fluorescens* (Jean‐Noël *et al*., 2012). Nevertheless, the calculations based on the Arrhenius model were in good agreement with actual cell counts in freeze‐dried lactic acid bacteria (King *et al*., 1998; Achour *et al*., 2001) and *Campylobacter jejuni* (Portner *et al*., 2007). An exact prediction by help of such models may not always be possible, especially if factors other than temperature, e.g. moisture, influence the degradation rate. Nevertheless, a good estimation may help excluding unsuitable conditions in a large‐scale screening process to narrow down the choice of parameters.

## Further considerations in approaching formulation needs

In addition to the core aspect of long‐term preservation, numerous other individual parameters are involved in the multistep process of formulating inoculants and add up to a literally infinite amount of possible approaches. This requires an elaborate and high‐throughput screening to narrow down the options. Several authors (Köhl *et al*., 2011; Slininger and Schisler, 2013; Segarra *et al*., 2015) suggest that this is best achieved using a stepwise approach, commencing with cheap, straightforward screening of a large range of variables and concluding with more sophisticated and labour‐intensive tests, such as glasshouse or field trials. At each step, only the parameters selected in previous steps are included (Köhl *et al*., 2011). This concept has been applied for identifying suitable process parameters for different strains of *P.fluorescens* (Slininger and Schisler, 2013; Schisler *et al*., 2016), *Enterobacter cloacae* (Slininger and Schisler, 2013) *Lysobacter capsici* (Segarra *et al*., 2015) and *P. phytofirmans* (Bejarano *et al*., 2017).

Early considerations prior to commencing with the actual formulation development need to take into account appropriate strain selection, which depends on the safety and efficacy of a bacterial agent and its ability to be mass‐produced. Furthermore, a broad host‐range, agricultural importance of the target crops and/or plant diseases as well as the availability of competing products are relevant for marketing ability and commercialization of the final product (Köhl *et al*., 2011; Slininger and Schisler, 2013). Efficacy should be known from experiments under controlled conditions, allowing for the determination of the required cell number in the final product. A possible dilution prior to field application (e.g. in tank mixes) as well as the total volume of inoculum delivered per individual plant (e.g. seed coating: depending on seed surface area) has to be considered. These aspects represent basic considerations that may decide over failure or success of the inoculant in its later stages of development.

From the manufacturers’ point of view, physical, chemical and biological consistency of the carrier material to establish routine processing is desirable (Stephens and Rask, 2000). Non‐toxicity to humans, plants and ecosystems (Catroux *et al*., 2001), biodegradability as well as the sustainable nature of raw materials and the production process need to be ensured (Bashan *et al*., 2014). Furthermore, the carrier should be sterile or suitable for simple sterilization to avoid contamination (Bashan *et al*., 2014). To increase acceptance by farmers, the formulation should be compatible with standard machinery, not associated with additional work steps and combinable with traditional techniques such as seed treatments (Catroux *et al*., 2001).

Also economic feasibility has to be taken into account. Expensive substances or fermentation protocols need to be excluded from the beginning and upscaling of the process should be prospective. Additives should be tested in a practical range of concentrations, taking into account limitations imposed by compatibility with machinery. This is relevant especially for foliar sprays. Assuming application rates of the active agent of 1–4 kg per ha and tank mixes of 1000 l per ha, Segarra *et al*. (2015) determined the feasible concentration of additives to be around 0.1% (w/v). Furthermore, protectants should be selected based on the expected stress factors occurring during formulation or application. For example, freeze‐dried inoculants require the incorporation of lyoprotectants, while products to be applied as a foliar spray demand UV protection (Segarra *et al*., 2015). All of these aspects can be clarified in a thorough research taking into account scientific literature, regulatory documents, manufacturer's technical instructions or consultation of experts from industry. Doing so prior to commencing with the actual, labour‐intensive formulation development is essential for achieving economic efficiency of the development process and to avoid dead‐end approaches.

## Future outlook

Numerous potent biocontrol and biofertilizer microorganisms, among them many non‐sporulating bacteria, are continuously being described, and the market of bacteria‐based products for application in agriculture is growing steadily (Ravensberg, 2015). In addition to scientific and technical aspects, legislative issues play a decisive role in practical implementation of products based on microorganisms.

### Regulatory framework

Aiming to promote sustainable technologies in agriculture, regulatory authorities are increasingly restricting the use of standard pesticides and simultaneously encouraging the implementation of alternatives (Lamichhane *et al*., 2017; Timmusk *et al*., 2017). In Europe, for example, legal frameworks such as the Directive 2009/128/EC are calling for a sustainable use of pesticides by enforcing restrictions such as the prohibition of their application in certain areas, and giving priority to non‐chemical methods wherever possible (European Parliament and Council of the European Union, 2009a). Existing legal frameworks are currently being revised and adapted to better address innovative agricultural techniques such as the application of microbial products. This is highly relevant, as present regulatory requirements are in many aspects a hindrance to successful commercialization of these products. For example, there is no clear definition of different terms in use, including biopesticide, biofertilizer and biostimulant. This results in uncertainties regarding the process of registration and authorization.

Generally, biopesticides are directed immediately against plant pests and comprise not only microorganisms, but also natural biochemicals or extracts (Baylis, 2016). Their mode of action implies that in Europe, Regulation (EC) No 1107/2009 on placing of plant protection products on the market (European Parliament and Council of the European Union, 2009b) provides the legal framework for their use. This regulation generally concerns active substances to (i) protect plants against pests and/or diseases, (ii) influence the life processes of plants except as nutrients, (iii) preserve plant products or (iv) destroy or prevent growth of undesired plants. The term ‘active substance’ explicitly comprises microorganisms. As a consequence, microbial biopesticides are regulated according to the chemical plant protection agents. Data required for their approval and use are set out in Commission Regulations (EU) 283/2013 (European Commission, 2013a) and (EU) 284/2013 (European Commission, 2013b). Data on their efficacy as well as safety (i.e. excluding toxicity to human health and non‐target organisms) have to be provided. However, the regulations are inexplicit as to how these data have to be generated and refer to test methods for chemical plant protection products or guidelines accepted by the competent authority (e.g. USEPA Microbial Pesticide Test Guidelines). This implies some uncertainty for the manufacturer as to how best to fulfil data requirements and which expenses to expect.

Efforts to speed up the approval of microbial biopesticides are currently being undertaken and discussed with stakeholders. For example, a draft for an amendment for Regulation (EC) 1107/2009 was issued and proposes the classification of microorganisms as low‐risk substances, thereby facilitating their registration and commercialization (European Commission, 2016).

In contrast to biopesticides, the term biofertilizer or biostimulant generally relates to an agent promoting plant growth and/or vigour by increasing nutrient availability, but precise definitions vary (Malusá and Vassilev, 2014). While standard inorganic fertilizers are regulated in Europe by the Regulation (EC) 2003/2003 (European Parliament and Council of the European Union, 2003), there is currently no legal framework setting out the commercialization and use of biofertilizer products. Instead, the member states provide national legislations regarding biofertilizers, which vary widely regarding important aspects such as definition, formulation and labelling (Baylis, 2016). To foster innovation in the field of fertilizers and to meet sustainability targets, the European Commission launched a revision of the existing regulations regarding fertilizers to extend their scope to fertilizers of biological origin (Traon *et al*., 2014). Acknowledging the need of a specific legislative framework for biofertilizers/biostimulants, the European Commission subsequently issued a proposal to amend existing regulations (European Parliament and Council of the European Union, 2016). It defines plant stimulant as ‘a product stimulating plant nutrition processes independently of the product's nutrient content with the sole aim of improving one or more of the following characteristics of the plant: nutrient use efficiency; tolerance to abiotic stress; crop quality traits’. Specifications for example regarding labelling and maximum contaminant levels are given. Furthermore, the proposal states that microbial plant biostimulants may exclusively contain *Azotobacter*,* Rhizobium*,* Azospirillum* spp. and/or Mycorrhizal fungi and display a minimum shelf life of six months. While the proposal was generally approved of by the European Biostimulants Industry Council (EBIC), the latter issues were criticized (European Biostimulants Industry Council, 2016). Arguing that the short positive list did not reflect the wide variety of biostimulant microorganisms available, the EBIC supports the approval of newly described microorganisms based on safety evaluation. Furthermore, instead of regulating the shelf life, the EBIC suggests to let the market decide. Thus, the proposed regulation has the potential to spur the implementation of microbial solutions, especially when adapted according to the suggestions made by the EBIC. This will open up new opportunities for many promising bacterial strains to enter the market – especially those with biostimulant action, many of which are Gram‐negative.

### Market development

The strong interest in sustainable, agricultural techniques is reflected in market figures. Sales of biopesticides have been increasing steadily, reaching a global value of around US$ 2.5 billion per year in 2016. Microbial products, particularly those based on *Bacillus thuringiensis*, constitute the most valuable category. Europe is the largest regional market with a share of 30%, whereas sales in North America and Asia Pacific account for 27% respectively and in Latin America for 13%. While biopesticides still account for a relatively small share of the total market of crop protection products (e.g. in Europe 4.2%), their value displays strong annual growth rates between 10 and 15% (2011–2016) (Baylis, 2016).

The EU pesticides database on active substances currently comprises 12 bacterial strains, most of which are spore‐forming (available from: ec.europa.eu/food/plant/pesticides/eu‐pesticides‐database/ 22/08/2017). The majority belongs to the genus *Bacillus* (*Bacillus firmus*,* Bacillus amyloliquefaciens*,* Bacillus pumilus*,* Bacillus subtilis and Bacillus thuringiensis*). Furthermore, two *Pseudomonas* species (*Pseudomonas chlororaphis* and *Pseudomonas* sp.) and two *Streptomyces* species (*Streptomyces lydicus* and *Streptomyces* K61) are approved. Globally, the amount of registered bacterial strains for plant protection is in the range of 77 (Ravensberg, 2015).

As opposed to biopesticides, the market situation of biostimulants is much less clear. This is due to variations in how biostimulants are defined and regulated in different countries, and the fragmented market, in which many small, local manufacturers are active (Watkins, 2016). Generally, bacteria from the genus *Bacillus*,* Pseudomonas* and *Rhizobium* are most frequently commercialized (Ravensberg, 2015). The sales of biostimulants were estimated at a total value of US$ 1.5 billion in 2015 and thus lower than the ones of biopesticides. However, they are growing at a similar rate of 10–12% per year (Watkins, 2016). These numbers underline the great potential for commercialization of products based on microorganisms.

## Conclusion

An essential step towards making use of diverse, potential plant beneficial strains is the development of suitable, strain‐specific formulations. To accelerate this labour‐intensive process, it is necessary to employ modern tools for monitoring of cell viability. Many of these tools are established in food processing industry or pharmaceutical research, but are not commonly used in formulation development of agriculturally relevant strains. It seems therefore very beneficial to adopt methods routinely applied in other fields of research. The same is true for drying methods. Furthermore, it is important to understand the interaction between bacteria and formulation materials. This requires an interdisciplinary, close collaboration between microbiologists, material scientists and agricultural scientists.

While using sophisticated scientific methods, the practical applicability – including material cost, production procedure and handling – should continuously be considered to allow for bacterial inoculants to take the step from the laboratory to the field.

## Conflict of interest

None declared.
